# Nanoparticles Engineered as Artificial Antigen-Presenting Cells Induce Human CD4^+^ and CD8^+^ Tregs That Are Functional in Humanized Mice

**DOI:** 10.3389/fimmu.2021.628059

**Published:** 2021-05-26

**Authors:** Sophia Giang, David A. Horwitz, Sean Bickerton, Antonio La Cava

**Affiliations:** ^1^ Department of Medicine, University of California Los Angeles, Los Angeles, CA, United States; ^2^ General Nanotherapeutics, Santa Monica, CA, United States; ^3^ Keck School of Medicine, University of Southern California, Los Angeles, CA, United States; ^4^ Department of Biomedical Engineering, Yale University, New Haven, CT, United States

**Keywords:** autoimmunity, T cells, T regulatory cells, immune tolerance, systemic lupus erythematosus, graft-versus-host disease, nanoparticles, humanized mice

## Abstract

Artificial antigen-presenting cells (aAPCs) are synthetic versions of naturally occurring antigen-presenting cells (APCs) that, similar to natural APCs, promote efficient T effector cell responses *in vitro*. This report describes a method to produce acellular tolerogenic aAPCs made of biodegradable poly lactic-co-glycolic acid (PLGA) nanoparticles (NPs) and encapsulating IL-2 and TGF-β for a paracrine release to T cells. We document that these aAPCs can induce both human CD4^+^ and CD8^+^ T cells to become FoxP3^+^ T regulatory cells (Tregs). The aAPC NP-expanded human Tregs are functional *in vitro* and can modulate systemic autoimmunity *in vivo* in humanized NSG mice. These findings establish a proof-of-concept to use PLGA NPs as aAPCs for the induction of human Tregs *in vitro* and *in vivo*, highlighting the immunotherapeutic potential of this targeted approach to repair IL-2 and/or TGF-β defects documented in certain autoimmune diseases such as systemic lupus erythematosus.

## Introduction

In natural conditions, the fate of activated T cells is determined by interaction between antigen-presenting cells (APCs) and T cells. This involves the engagement of the MHC/peptide complex on the APC and the T-cell receptor (TCR) on the T cell, and the complementary costimulatory molecules on the APC and the T cell. The modulation of this critical step of the adaptive immune response can have very important immunotherapeutic implications in the clinic for the generation of effector and regulatory immune responses. However, the fine tuning of APCs interactions with T cells for therapeutic purposes has been difficult to achieve. The natural sources of APCs are scarce. Large amounts of starting cells are required for enrichment and/or sorting, and technical procedures for their preparation are costly and time-intensive. For these reasons, alternative procedures have been developed that include engineering cellular and acellular artificial APCs (aAPCs) (1). Cellular aAPCs are engineered from primary cells or from transformed (human or xenogeneic) cells using retroviral or lentiviral vectors to express the desired costimulatory molecules and adhesion molecules for the expansion and/or long-term growth of functional T cells (2). Other cellular aAPCs may also express human HLA molecules to generate antigen-specific cells for patients with a given HLA (3). Those cellular aAPCs that carry the necessary components for interaction and engagement of the cell surface ligands on T cells for activation and proliferation (4, 5) have been used in cancer immunotherapy and immunization in infection because of their ability to generate effector T cell responses. However, their pro-inflammatory activity makes them unsuitable in settings where effector immune responses are deleterious to the host, such as in autoimmunity.

We report here the development of acellular aAPCs that target T cells and induce them to become functional Tregs *in vitro* and *in vivo*. We had previously shown that nanoparticle (NP)-mediated delivery of IL-2 and TGF-β (two cytokines that are deficient in SLE) to mouse CD2^+^ and CD4^+^ cells induced tolerogenic immune responses that protected mice from a lupus-like syndrome (6, 7). Here we extend those results and show that we can induce human functional CD4^+^ and CD8^+^ Tregs that suppress xenogenic graft-versus-host disease (GvHD) in humanized mice using aAPC NPs, providing a proof-of-principle of immunotherapeutic restoration of immune homeostasis in conditions of immune dysregulation associated with chronic inflammation.

## Methods

### Preparation of PLGA Nanoparticles

Poly lactic-co-glycolic acid (PLGA) NPs were prepared as described elsewhere (6). After preparation, the NPs were characterized through examination of physical properties, encapsulation metrics, and release kinetics according to standard procedures (6). By dynamic light scattering, NPs were found to have a mean ± SD hydrodynamic diameter of 245 ± 2 nm with a low polydispersity index indicative of a uniform NP population with a relatively tight size distribution. Cytokine encapsulation was measured by ELISA after NPs were disrupted using DMSO, and standard curves were generated using cytokine standards with all wells supplemented to contain 5% volume/volume DMSO and the appropriate concentration of empty NPs. NPs contained a mean ± SD of 7.4 ± 0.4 ng TGF-β and 1.9 ± 0.1 ng IL‐2 per mg of NP. For cell targeting, NPs diluted in PBS were incubated 10 minutes prior to use with the relevant biotinylated targeting antibody (anti-CD4, -CD8 or -CD3) at a concentration ratio of 2 μg antibody/mg NP.

### Preparation of Human PBMCs

Human peripheral blood mononuclear cells (PBMCs) were prepared from heparinized venous blood of healthy adult volunteers by Ficoll-Hypaque density gradient centrifugation and used fresh (for transfer experiments) or cultured for 5 days in U-bottom well plates at a concentration of 0.5 x 10^6^/well in complete AIM V™ medium (Thermo Fisher Scientific, Waltham, MA). All protocols that involved human blood donors were approved by the IRB at the University of California Los Angeles. In some experiments, PBMCs were cultured with anti-human CD3/CD28 Dynabeads (Thermo Fisher Scientific) or with IL-2 (100 U/ml) and TGF-β (5 ng/ml) or anti-TGF-β (1D11) (all from R&D Systems, Minneapolis, MN). *In vitro* suppression assays were performed according to standard protocols (6). CD4^+^CD25^-^ T cells isolated by negative selection to a purity of >95% using the Miltenyi Biotec CD4^+^CD25^+^CD127^dim/-^ Regulatory T Cell Isolation kit II served as responder cells in cocultures for 3 days with autologous Tregs (positive fraction) isolated with the same kit, following the manufacturer’s instructions. Culture supernatants were analyzed for IFN-γ content by ELISA (R&D Systems). Proliferation was evaluated by a liquid scintillation counter following addition of ^3^H‐thymidine (1 μCi/well) 16 hours before analysis.

### Flow Cytometry

Human PBMCs or magnetic-bead sorted cells were stained following standard procedures with the following FITC-, PE-, PerCP- or APC-conjugated anti-human antibodies: CD4 (RPA-T4), CD8 (RPA-T8), CD25 (MEM-181), CD127 (eBioRDR5), FoxP3 (PCH101), CD122 (TU27), CD45RA (HI100), or isotype controls. All antibodies were from Thermo Fisher Scientific. Data were acquired on a FACSCalibur™ flow cytometer (BD Biosciences, San Jose, CA) and analyzed using FlowJo™ software (BD, Franklin Lakes, NJ).

### Mice

To assess the functional properties of the human Tregs induced by aAPCs, we used the human-anti-mouse xenogeneic GvHD model. The disease develops in recipient NOD/*scid*/*IL2r* common γ chain^−/−^ (NSG) mice following the transfer of human PBMCs (8) and, like human lupus, these mice develop B cell hyperactivity and increased IgG production. NSG mice were purchased from the Jackson Laboratory (Bar Harbor, ME) and housed under specific pathogen-free conditions in microisolator cages with unrestricted access to autoclaved food and sterile water. 10^7^ fresh human PBMCs were resuspended in 200 µl of PBS in insulin syringes and injected i.v. *via* the tail vein into individual unconditioned NSG mice of 8-12 weeks of age. The mice also received i.v. (individually) 1.5 mg IL-2/TGF-β-loaded NPs decorated with anti-CD3 (OKT3, Thermo Fisher Scientific), starting on the day of transfer of human PBMCs, according to a previously described protocol (6): day 0, 3, 6, 9, 12. Control mice received empty uncoated NPs or PBS under identical conditions as the above NP-treated mice. The experiments were performed according to the guidelines of the Institutional Animal Committee of the University of California Los Angeles. Animals that developed hunched posture combined with lethargy and/or lack of grooming, reduced mobility or tachypnea, were euthanized and an end-point of survival was recorded at the time of sacrifice. Disease was monitored using a validated scoring system (9) that evaluates each of the five following parameters as 0 if absent or 1 if present: 1) weight loss >10% of initial weight; 2) hunching posture; 3) skin lesions (patchy alopecia); 4) dull fur; 5) diarrhea. Dead mice received a total score of 5 until the end of experiment. Peripheral blood (to separate PBMCs for flow cytometry) and plasma were collected on days 0, 4, 14, 21 and 50. Plasma concentrations of human IgG were measured by ELISA (Thermo Fisher Scientific). For histologic evaluations, lung, liver and colon were collected on day 50 after the transfer of PBMCs. Tissues were fixed in formalin, paraffin embedded, and sections stained with hematoxylin/eosin.

### Statistical Analyses

Assessment for normal distribution was done by Shapiro-Wilks test. Comparisons between two groups were evaluated using (post-hoc) Student’s *t* test; comparisons among multiple groups used one-way ANOVA with Bonferroni’s correction. Differences in Kaplan-Meier survival curves were analyzed by the log-rank test. Data were analyzed using GraphPad Prism software; *P *values of <0.05 were considered significant.

## Results

### Use of NPs as Acellular aAPCs to Induce CD4^+^CD25^hi^FoxP3^+^CD127^-^ and CD8^+^FoxP3^+^ T Cells

We recently reported that NPs loaded with IL-2 and TGF-β and targeted to T cells inhibited the production of anti-DNA autoantibodies in (C57BL/6 × DBA/2)F_1_ (BDF1) hybrid mice that develop lupus-like disease following the transfer of splenocytes from parental DBA/2 mice (6). Specifically, the NPs promoted a switch to tolerogenic responses with an induction of CD4^+^ and CD8^+^ Tregs that were responsible for the mitigation of disease manifestations and prolonged the survival of BDF1 lupus mice.

**Figure 1:**
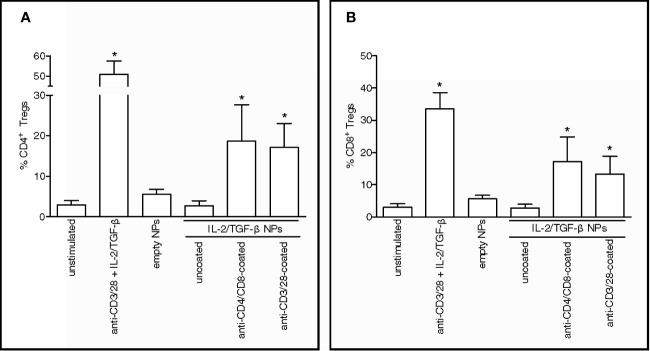
Nanoparticles (NPs) loaded with tolerogenic cytokines and targeted to human T cells induce CD4^+^ and CD8^+^ Tregs *in vitro*. PBMCs from healthy volunteers (n = 5) were cultured for 5 days in the presence of 100 µg/ml NPs loaded with IL-2 and TGF-β that had been either left uncoated or decorated with antibodies to T cells (anti-CD4/CD8 or anti-CD3/28). Cultures with medium only and either no NPs (unstimulated) or NPs kept unloaded (empty) served as negative controls; cultures with anti-CD3/28 beads at a ratio of 0.2 beads/cell in the presence of soluble IL-2 and TGF-β served as positive control. Results show increased numbers of CD4^+^CD25^hi^CD127^-^FoxP3^+^ **(A)** and CD8^+^FoxP3^+^ T cells **(B)** in the presence of NPs decorated with anti-CD4/CD8 Ab or anti-CD3/CD28 Ab. *P < 0.05 by the Student’s *t* test in the comparison with unstimulated cells.

To extend those findings to humans, PBMCs from healthy donors were incubated with PLGA NPs loaded with IL-2 and TGF-β and targeted to CD4^+^ and CD8^+^ T cells. The observed expansion *in vitro* of CD4^+^CD25^hi^FoxP3^+^CD127^-^ (**Figure 1A**) and CD8^+^FoxP3^+^ T cells (**Figure 1B**) after NP-mediated delivery of tolerogenic cytokines to human CD4^+^ and CD8^+^ T cells suggested the induction of immunoregulatory cells (10–14).

### Paracrine Delivery of Cytokines to Human T Cells by aAPC NPs Leads to the Induction and Expansion of Functional Tregs

These findings led us to wonder whether the NPs could induce a tolerogenic T-cell program by acting as acellular aAPCs that delivered engage the TCR rather than CD4 or CD8 and deliver IL-2 and TGF-β. We found that the NPs loaded with tolerogenic cytokines and coated with anti-CD3/28 antibodies to trigger TCR stimulation efficiently expanded CD4^+^CD25^hi^FoxP3^+^CD127^-^ (**Figure 1A**) and CD8^+^FoxP3^+^ (**Figure 1B**) T cells *in vitro*, indicating the ability of NPs to operate as acellular aAPCs capable to induce human T cells with an immunoregulatory phenotype.

**Figure 2:**
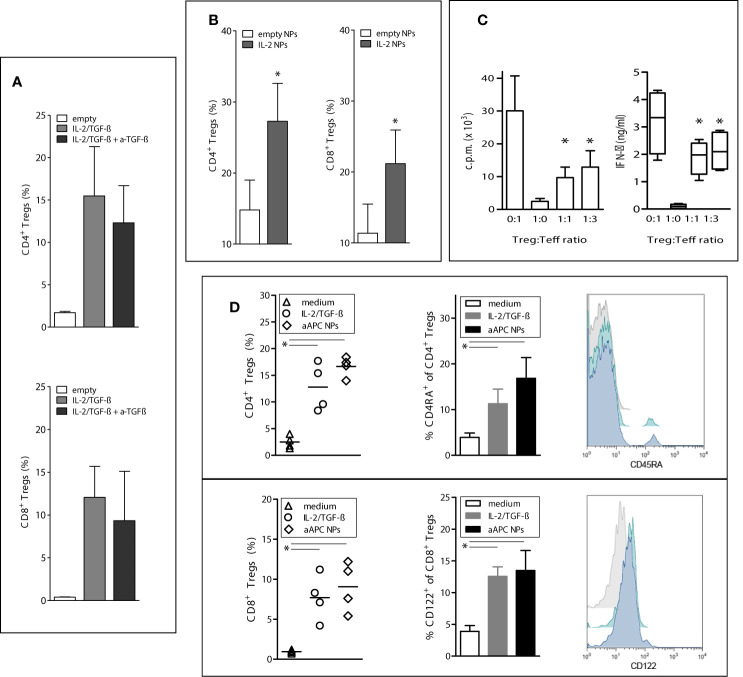
Paracrine delivery of cytokines by aAPC NPs to human T cells favors the induction and expansion of functional Tregs *in vitro*. To induce Tregs, human PBMCs from healthy donors (n = 6) were cultured with IL-2/TGF-β-loaded NPs targeted to T cells (decorated with anti-CD3/28) for 5 days. To study the expansion of the induced Tregs, the cultured cells were then washed twice before incubation with IL-2-encapsulating NPs or empty NPs for 5 more days with or without anti-TGF-β antibody (10 µg/ml). **(A)** The expansion of aAPC NP-induced CD4^+^ and CD8^+^ Tregs is not influenced significantly by the blockade of TGF-β. P not significant in the presence or absence of anti-TGF-β. **(B)** T-cell-targeted NPs that only encapsulate IL-2 promote the expansion of CD4^+^ and CD8^+^ Tregs; *P < 0.05 by the Student’s *t* test. **(C)** CD4^+^ Tregs induced by aAPC NPs targeted to T cells suppress *in vitro* the proliferation (left) and IFN-*γ* production (right) of cocultured CD4^+^CD25^-^ T cells. *P < 0.05 in the comparison with Treg : Teff at the 0:1 ratio (only stimulated T effector cells). **(D)** Comparison of induction of CD4^+^ (top) and CD8^+^ (bottom) Tregs by aAPC NPs decorated with anti-CD3/28 vs. anti-CD3/28 beads in the presence of soluble IL-2/TGF-β. Control cultures had medium only. Percentage numbers and representative histograms (grey, medium only; green, IL-2/TGF-β; blue, aAPC NPs) for the expression of CD45RA by CD4^+^ Tregs and CD122 by CD8^+^ Tregs (n = 4 donors). *P < 0.05 by the Student’s *t* test in the comparison vs. medium only; not significant between cultures with IL-2/TGF-β and aAPC NPs.

Having found that the delivery of IL-2 and TGF-β to T cells by the NPs allowed human T cell differentiation into CD4^+^CD25^hi^FoxP3^+^CD127^-^ and CD8^+^FoxP3^+^ T cells, we investigated the temporal contribution of TGF-β to the process. The presence of TGF-β was required for the induction of CD4^+^ and CD8^+^ Tregs but was dispensable for their expansion since its blockade did not influence expansion (**Figure 2A**). Both IL-2 and TGF-β were required for the induction but IL-2 alone could promote expansion (**Figure 2B**). Thus, the IL-2 and TGF-β delivered by the aAPC NPs play different roles in the generation of the human Tregs, being both required for induction but being only IL-2 required for expansion.

The activity of the aAPC NP-induced cells was confirmed by assays of *in vitro* suppression that indicated that the aAPC NP-induced CD4^+^ Tregs efficiently suppressed proliferation and production of proinflammatory cytokines from T effector cells (**Figure 2C**). Of note, aAPC NPs induced similar numbers of both CD4^+^ and CD8^+^ Tregs (10–14) as compared to those standardly induced by incubation with IL-2 and TGF-β (6, 15) (**Figure 2D**). Moreover, the incubation with aAPC NPs increased the expression of CD122 on CD8^+^FoxP3^+^ cells and CD45RA on CD4^+^FoxP3^+^ cells (**Figure 2D**). The interest of this finding lies in the fact that CD122 is a classical CD8^+^ Treg marker (15), while CD45RA^+^ CD4^+^ Tregs are valuable for adoptive transfer of CD4^+^ Tregs in immune-mediated disorders since they maintain FoxP3 expression and retain homing receptors (CD62 and CCR7) after extensive proliferation (16).

### Induction of Tregs *In Vivo* by aAPC NPs Associates With the Protection of Humanized NSG Mice From Lupus-Like Disease

**Figure 3:**
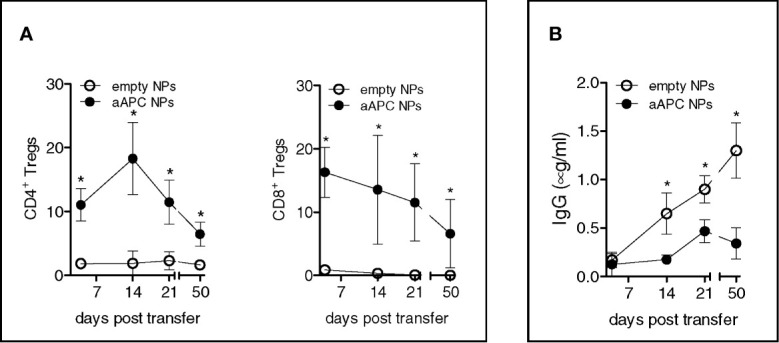
Humanized NSG mice treated with aAPC NPs have increased numbers of circulating CD4^+^ and CD8^+^ Tregs and reduced levels of human IgG. Individual NSG mice (n = 12) that received i.v. 10^7^ human PBMCs each for the induction of lupus-like disease were divided into two groups of 6 mice each. One group received IL-2/TGF-β-loaded NPs targeted to T cells (decorated with anti-CD3 Ab), the other group that served as control received empty, untargeted NPs according to the protocol detailed in the *Methods*. **(A)** PBMCs were analyzed *ex vivo* by flow cytometry at the time points indicated. Relative frequency of peripheral CD4^+^ and CD8^+^ Tregs was derived from counts of CD4^+^ and CD8^+^ T cells. **(B)** Human IgG antibodies in plasma were measured by ELISA. *P < 0.05 by the Student’s *t* test.

Since the suppressive activity of the Tregs *in vitro* might not necessarily correlate with a suppressive activity *in vivo (* 17), we evaluated the relevance of the above *in vitro* findings to *in vivo* settings. Taking advantage of the known protective effects of Tregs in allograft rejection, we tested the immunotherapeutic potential of the aAPC NPs in a mouse model of human-anti-mouse GvHD (which reproduces manifestations of lupus-like disease *in vivo*) (8). Individual NSG mice received i.v. 10^7^ human PBMCs to develop GvHD. One group concomitantly received (anti-CD3 Ab-) T-cell targeted NPs encapsulating IL-2/TG-β, one control group received empty uncoated NPs, and another control group only received vehicle (PBS). The results showed that the mice that received T-cell targeted NPs encapsulating IL-2/TG-β had an *in vivo* expansion of both CD4^+^ and CD8^+^ Tregs that was absent in mice that had received empty NPs or no NPs (**Figure 3A**) and that associated with a reduction in the levels of circulating human IgG (**Figure 3B**). Of note, the expansion of the Tregs remained above baseline levels throughout the experiment until its termination at day 50 (**Figure 3A**).

**Figure 4:**
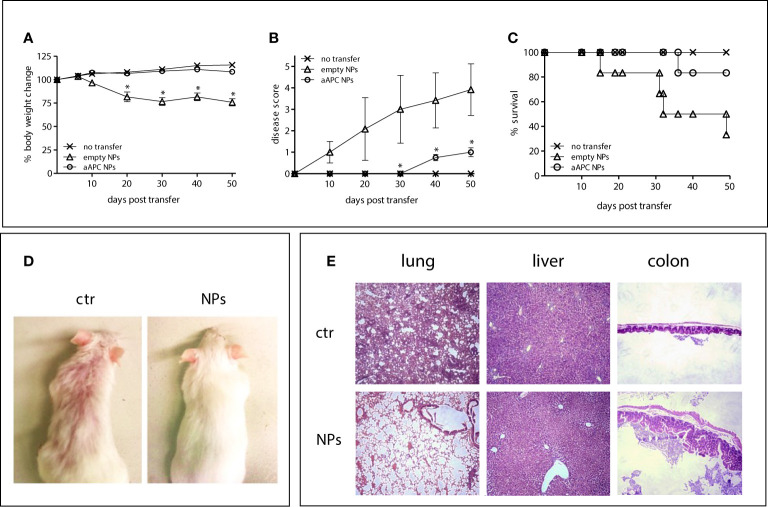
aAPC NPs engineered to induce Tregs *in vivo* suppress lupus-like disease in NSG mice transferred with xenogenic human PBMCs. Six NSG mice per group were left untreated (no transfer) or injected with human PBMCs together with PBS (ctr) or aAPC NPs (see *Methods*). **(A–C)** Body weight change **(A)**, clinical disease score **(B)** and Kaplan-Meier survival curves **(C)** at the time points indicated on the *x* axis. *P < 0.05 (aAPC NPs vs controls). **(D, E)** Comparisons between mice receiving aAPC NPs (NPs) or not (ctr) for skin appearance **(D)** and tissue histopathology of lung, liver and colon (20 x magnification) **(E)**.

Importantly, the NSG mice that received T-cell targeted NPs loaded with IL-2/TGF-β had significantly reduced disease manifestations. The aAPC NP-protected mice did not lose weight after the transfer of human PBMCs (**Figure 4A**) and had an extended survival (**Figure 4B**) as compared to the mice that had not received NPs or that had received empty NPs (**Figures 4A, B**). Mice treated with NPs also had reduced human IgG levels (**Figure 4C**) and improved skin morphology (**Figure 4D**). Finally, the histopathology of lung, liver and colon of the NSG mice receiving aAPC NPs showed a significant protection as compared to the control mice (**Figure 4E**). These results provide evidence that the aAPC NPs induced therapeutic Tregs.

## Discussion

We show that PLGA NPs can be used as acellular aAPCs for *in vitro* and *in vivo* expansion of functional human Tregs that suppress xenogeneic graft-versus-host disease. In the *in vitro* experiments with human cells, the aAPC NPs containing IL-2 and TGF-β were decorated with anti-CD3/CD28. In the *in vivo* experiments, the NPs were decorated with anti-CD3 Ab only since human T cells were activated by the mouse MHC antigens. In both cases CD4^+^CD25^hi^CD127^lo^FoxP3^+^ and CD8^+^FoxP3^+^ Tregs were generated and expanded. While IL-2 and TGF-β were required for Treg induction, only IL-2 was required for Tregs expansion.

When APCs engage the TCR through the MHC/antigen complex and provide costimulatory signals to T lymphocytes, cell differentiation and functional activation ensue. The replication of this process by aAPCs - used as synthetic platforms - can recapitulate the natural interaction between APCs and T cells, allowing the delivery of signals to T cells and the initiation of adaptive immune responses (5, 18) that include a paracrine delivery of IL-2 to T cells (as in our aAPCs). Our strategy of employing aAPCs that encapsulated a payload for the promotion of tolerogenic immune responses could represent a new tool with immunotherapeutic potential, being effective in humanized mice. The fact that PLGA is biocompatible and has a favorable safety profile in clinical settings envisions the possibility of a rapid translational potential of this approach to the clinic (19).

The expansion of human Tregs with aAPC NPs has the advantage of limiting the deleterious effects associated with an *in vivo* induction of Tregs achievable through systemic treatments with cytokines that carry non-targeted actions. We acknowledge that our NPs did not include components of antigen specificity, differently from the paramagnetic iron-dextran NPs that expressed peptide/MHC and anti-CD28 antibodies and were used in organ-specific autoimmune diseases (20, 21). We believe that the induction of polyclonal Tregs might be advantageous in conditions such as SLE, where the chronic systemic autoimmune response to multiple self-antigens (22) benefits from polyclonal Tregs (23). We also think that the expansion of both CD4^+^ and CD8^+^ Tregs *in vivo* in SLE will have more beneficial effects than that of each subset alone because of the protective activity of CD8^+^ Tregs in the disease (15). However, a limitation of our study is that it does not distinguish the relative contribution of the CD4^+^ and CD8^+^ Tregs in the observed *in vivo* protective effects. Since in previous work with CD4^+^ and CD8^+^ Tregs induced *ex vivo* the CD8^+^ Tregs were major contributors to the protection of immunodeficient mice from human anti-mouse GvHD through non-cytotoxic suppressive effects on allogeneic cells (24), we suggest that the CD8^+^ Tregs in our current study did contribute to the protective effects on the human anti-mouse GvHD. The extent needs to be investigated directly.

Multiple possible therapeutic applications can benefit from the utilization of Tregs yet several problems hamper practical use. In general, the small number of Tregs that circulate in the peripheral blood requires Tregs expansion *ex vivo* before infusion in sufficient numbers. This associates with significant costs and specific technical requirements (25). Additionally, repeated treatments for the patient are often required, since *ex vivo*-expanded Tregs can become instable over time (26). Finally, chronic inflammation in autoimmune patients promotes the reversal of the phenotype of the transferred Tregs into T effector cells (27), and Treg potency may decrease over time (28).

Here we report that aAPC NPs can sustain Treg activity with prolonged efficacy in humanized mice, providing rational grounds for an immunotherapeutic expansion *in vivo* of human Tregs for the suppression of proinflammatory responses. Future investigations will address whether the inclusion of antigen-specificity in the aAPC NPs can further improve their tolerogenic benefits.

## Data Availability Statement

The raw data supporting the conclusions of this article will be made available by the authors, without undue reservation.

## Ethics Statement

The studies involving human participants were reviewed and approved by Institutional Review Board of the University of California Los Angeles. Written informed consent for participation was not required for this study in accordance with the national legislation and the institutional requirements. The animal study was reviewed and approved by the Animal Research Committee of the University of California Los Angeles.

## Author Contributions

ALC and DH designed the study. SG, SB, and ALC performed experiments. SG, DH, and ALC analyzed and discussed the results and gave critical intellectual contributions. ALC wrote the manuscript. DH contributed to its final editing. All authors contributed to the article and approved the submitted version.

## Funding

Supported in part by the NIH grants HD097531 and AI154935 to ALC and the NIH grant GM007205 to SB.

## Conflict of Interest

DH owns stocks and stock options at General Nanotherapeutics and Toralgen, Inc. DH has filed a US patent application (63118863) on the methods described in this manuscript to produce tolerogenic NPs for the treatment of immune-mediated diseases.

The remaining authors declare that the research was conducted in the absence of any commercial or financial relationships that could be construed as a potential conflict of interest.
